# Therapeutics

**Published:** 1878-02

**Authors:** 


### THERAPEUTICS.

BISMUTH FOR PROLAPSUS ANI AND HEMORRHOIDS.—Dr. Cland. (*In Clinica di Napoli and La France Médicale*, Oct 27, 1877.) The treatment consists in introducing into the intestine every day, after reduction of the prolapsus, a teaspoonful of powdered bismuth, moistened with a little water, and mixed with powdered starch.

Good results have been obtained from the same treatment in prolapsus in children, and in grave cases of hemorrhoids.

The Action of Pilocarpin.—Dr. E. Kurz (*Memorabilien*, 1877, No. 11). A patient with emphysema of the lungs and severe bronchial catarrh, had œdema of the lower extremities, atheromatous arteries, feeble and irregular cardiac sounds, and a serious dyspnœa. Half a drachm of a two per cent. solution of pilocarpin was injected under the skin. After ten minutes the medicine began to show its specific effect. The perspiration, however, was not very profuse; the skin became moist, but the sweat did not collect in drops. The salivation was very pronounced, and the bronchial mucus seemed to be expectorated with greater ease. The excretion of urine was not increased. The œdematous swelling of the legs diminished at once. The dyspnœa was less severe, and the patient, who had neither headache nor nasea, felt quite comfortable. The remedy was repeated several times, always with the same pleasant effect upon the patient's condition.

Elastic Crayon of Nitrate of Silver.—Pagot (*Annales de Gynécologie and Gazette Obstét.*, No. 21).

M. Pajot takes a laminaria tent two millimetres in diameter, dips it in thick mucilage, and then rolls it in finely powdered fused nitrate of silver, and allows it to dry. He thus obtains an elastic crayon of the ordinary size, which may be introduced into the uterus without fear of breaking. He believes this means to be applicable to other cavities, and for other more powerful caustics.

Pomade for Porrigo.—Maccomac. (*L' Union Médicale*, Nov. 2d.)

| | | |
|---|---|---|
| Petroleum | - - - - | 15 grammes. |
| Prepared lard | - - - - | 30 " |
| Essential oil of lavender | - - | q. s. |

Mix.

To be softened and spread, by means of a soft brush, on the scalp, previously shaved. Before a new application, the first should be removed by means of warm water and soft soap.

This preparation is useful in tinea favosa, and in itch, and for lice of head, body, or pubis.

Antiherpetic Mercurial Lotion.—(*L' Union Médicale*, Dec. 15, 1877.)

| | | |
|---|---|---|
| Bichloride of mercury - - | 0.10 | grammes. |
| Chloride of ammonium - | 2. | " |
| Alcohol - - - - | 15. | " |
| Distilled water of bitter almonds | 15. | " |

Dissolve the salts in the alcohol and almond water, and add,

| | | |
|---|---|---|
| Emulsion of bitter almonds - | 500. | grammes. |

For lotions in pityriasis, acne, chronic eczema, and pruritus.

Sulphur Lotion.—(*L' Union Médicale*, Nov. 29, 1878.)

| | | |
|---|---|---|
| Sodium Sulphide - - - | 15 | grammes. |
| Distilled water - - - - | 150 | " |

Dissolve.

A tablespoonful in a quart of very warm water, for lotions for the heads of children suffering with crusta lactea, and for the face in moist eruptions.

It may also be used with success in chronic eczema of the nostrils, and for puritus vulvæ.

Borax and Nitrate of Potassium in Sudden Hoarseness.—Dr. Corson. (*Gaz. Med. Italiana and La France Méd.*, Oct. 27, 1877.) The author recommends singers, speakers, and others, who find themselves suddenly hoarse, to dissolve slowly in the mouth a piece of borax the size of a pea. This provokes an abundant flow of saliva, which moistens the mouth and throat. This local action should be aided by an equal dose of nitrate of potassium dissolved in a small glass of very warm water at bed-time.

Liquorice in Diabetes Mellitus.—M. Martin (*Bulletin Gén. de Thérap.*, September, 1877, *Phil. Med. Times*) has experimented with liquorice in order to determine whether it can be employed in the dietetics of diabetic patients. Having under his care a man suffering with this disorder, he made him drink daily about one quart of an infusion of liquorice root, and ordered his coffee to be sweetened with a small quantity of a stronger infusion. This lessened the bitterness of the coffee, but did not destroy its aroma or other qualities. A daily examination of the urine showed not the least increase in the amount of sugar excreted. These experiments, and others by the same author, show that patients of this description may use liquorice without fear of increasing their malady, for the purposes for which sugar is ordinarily employed.

ANTIDIARRHŒIC INJECTION.—Bouchut. (*Gazette Obstétricale*, Nov. 5, 1877.)

| | | |
|---|---|---|
| Borate of soda - - | 10, 15, and 20 | grammes. |
| Water - - - - - - | 125 | " |

Dissolve.

This is advised for nervous or catarrhal diarrhœas in children; diarrhæas which may cause death without leaving any appreciable lesion. The author was led to employ borax, from the success which follows its use in the diseases of the buccal mucous membrane. He thinks it acts as a feeble astringent, and at the same time as an alkali capable of neutralizing the acidity of the liquids in the large intestine. In any case borax has the advantage of not being irritant like nitrate of silver.

CHLORATE OF POTASSIUM IN CERTAIN FORMS OF DIARRHÆA. It is stated (*La Andalusia Medica*, Cordova, August, 1877, *Phil. Med. Times*) that Dr. Vonfigli employs chlorate of potassium in the diarrhœas which occur chiefly in cachectic patients attacked with nervous affections, and which consist of very frequent serous evacuations. These diarrhœas, called by the author "vaso-paralytic," are rebellious to treatment by astringents and narcotics, and may be the percursor of death. Experiments have shown that chlorate of potassium increases the contractility of the muscular coat of the vessels, and hence the indication for its use. To obtain the favorable results stated, the drug must be continued for a long time, and in severe cases increased in dose. The dose varies from two to ten grains in the twenty-four hours, according to the individual case. The author thinks that by analogy this treatment ought to be favorable in the diarrhœa of old age, in cholera, and in certain serous diarrhœas of hot countries.

